# *Saccharomyces cerevisiae *FKBP12 binds *Arabidopsis thaliana *TOR and its expression in plants leads to rapamycin susceptibility

**DOI:** 10.1186/1471-2229-7-26

**Published:** 2007-06-01

**Authors:** Rodnay Sormani, Lei Yao, Benoît Menand, Najla Ennar, Cécile Lecampion, Christian Meyer, Christophe Robaglia

**Affiliations:** 1 DSV-DEVM Laboratoire de Génétique et de Biophysique des Plantes, UMR 6191 CNRS-CEA-Université de la Méditerranée, Faculté des Sciences de Luminy,163 Avenue de Luminy, 13009 Marseille France; 2 Beijing Agro-Biotechnology Research Center, Beijing Academy of Agriculture and Forestry Sciences. P.O. Box 2449, 100097 Beijing, China; 3 Cell & Developmental Biology Department, John Innes Centre, Norwich Research Park, Colney, Norwich, Norfolk, NR4 7UH, UK; 4 Unité de Nutrition Azotée des Plantes, Institut Jean-Pierre Bourgin (IJPB) INRA 78026 VERSAILLES Cedex, France

## Abstract

**Background:**

The eukaryotic TOR pathway controls translation, growth and the cell cycle in response to environmental signals such as nutrients or growth-stimulating factors. The TOR protein kinase can be inactivated by the antibiotic rapamycin following the formation of a ternary complex between TOR, rapamycin and FKBP12 proteins. The TOR protein is also found in higher plants despite the fact that they are rapamycin insensitive. Previous findings using the yeast two hybrid system suggest that the FKBP12 plant homolog is unable to form a complex with rapamycin and TOR, while the FRB domain of plant TOR is still able to bind to heterologous FKBP12 in the presence of rapamycin. The resistance to rapamycin is therefore limiting the molecular dissection of the TOR pathway in higher plants.

**Results:**

Here we show that none of the FKBPs from the model plant Arabidopsis (AtFKBPs) is able to form a ternary complex with the FRB domain of AtTOR in the presence of rapamycin in a two hybrid system. An antibody has been raised against the AtTOR protein and binding of recombinant yeast ScFKBP12 to native Arabidopsis TOR in the presence of rapamycin was demonstrated in pull-down experiments. Transgenic lines expressing ScFKBP12 were produced and were found to display a rapamycin-dependent reduction of the primary root growth and a lowered accumulation of high molecular weight polysomes.

**Conclusion:**

These results further strengthen the idea that plant resistance to rapamycin evolved as a consequence of mutations in plant FKBP proteins. The production of rapamycin-sensitive plants through the expression of the ScFKBP12 protein illustrates the conservation of the TOR pathway in eukaryotes. Since AtTOR null mutants were found to be embryo lethal [1], transgenic ScFKBP12 plants will provide an useful tool for the post-embryonic study of plant TOR functions. This work also establish for the first time a link between TOR activity and translation in plant cells

## Background

The TOR (Target Of Rapamycin) pathway is a conserved eukaryotic pathway regulating growth, cell integrity and survival as a function of many different inputs including nutrient availability, energy status and mitogens in multicellular organisms [2-4]. TOR is a very large protein with a Ser/Thr kinase domain preceded by several HEAT repeats which interact with the numerous TOR protein partners. Studies in yeast and animal cells have shown that TOR acts positively on the activity of the eIF4F translation initiation complex and on the transcription of ribosomal RNA and protein genes therefore promoting growth in nutrient sufficient conditions [5-7]. In starvation conditions TOR regulates the utilization of alternative energy resources, allows autophagy and generally drive the cell towards survival pathways [8-12]. Rapamycin, an antibiotic produced by the soil bacteria *Streptomyces hygroscopicus *was found to mimic starvation responses in yeast through TOR inactivation and cell cycle arrest in G1 [13]. Rapamycin leads to the formation of a ternary complex by binding simultaneously to the FRB [FKP12 and Rapamycin Binding] domain of TOR and to the ScFKBP12 protein [14]. ScFKBP12 is a peptidylprolyl isomerase that was originally identified as the cytosolic receptor for the immunosuppressive drugs FK506 and rapamycin [15]. This ternary complex is inactivating the TOR kinase activity in a specific manner since no other cellular targets of rapamycin are known [16]. In animal cells, rapamycin has been shown to promote the dissociation of the TOR/Regulatory Associated Protein of TOR [RAPTOR] complex [17]. RAPTOR, a member of one of the two TOR [TORC1] complexes, is supposed to recruit the various TOR substrates [18-20].

Arabidopsis possesses a single TOR encoding gene and its inactivation was found to arrest embryo developement at an early stage [1]. Further studies demonstrated that *AtTOR *expression is limited to regions where cell proliferation occurs such as apical and root meristematic zones. Two homologs of Raptor have been found in Arabidopsis [21,22]. Some targets of TOR, such as eIF4E and S6 ribosomal kinase (S6K) are also conserved in plants [23,24] and plant TOR was found to phosphorylate S6K [25].

Rapamycin susceptibility is widespread among eukaryotes since the growth of most fungi and animal cells is affected by rapamycin. Although lands plants where found to be resistant to rapamycin action, green algae, such as *Chlamydomonas reinhardtii *are susceptible to rapamycin [11].

Examination of the amino acid sequence of Arabidopsis FKBP12 protein shows that several amino acids known to be important for rapamycin binding in yeast an animal FKBP12s are replaced which suggests that susceptibility to rapamycin has been lost during land plant evolution due to the inability of plant FKBP12 to bind rapamycin and to promote the formation of the TOR inactivation complex [11]. To support this hypothesis, expression of *Vicia faba *FKBP12 did not restore the sensitivity of a yeast ScFKBP12 mutant to rapamycin [26]. This result was further strenghtened by the observation that, in two-hybrid interaction experiments in yeast, the conserved FRB domain of AtTOR was able to bind to ScFKBP12 in a rapamycin dependent manner while it did not binds to AtFKBP12 [1]. Furthermore, interaction between the AtTOR FRB domain and human FKBP12 was also described [25]. The experiments described above therefore led us to the hypothesis that rapamycin susceptibility in plants could be restored by the expression of an heterologous FKBP protein. This would allow the use of rapamycin in plants to decipher the outputs of the TOR signaling pathway and to analyze the consequences of a post-embryonic inactivation of AtTOR. In this work we show that native AtTOR binds *in vitro *to recombinant ScFKBP12 in the presence of rapamycin and that expression of ScFKBP12 in transgenic plants results in a partial and rapamycin-dependent arrest of root growth.

## Results

### AtFKBP proteins cannot interact with rapamycin and TOR

**Figure 1:**
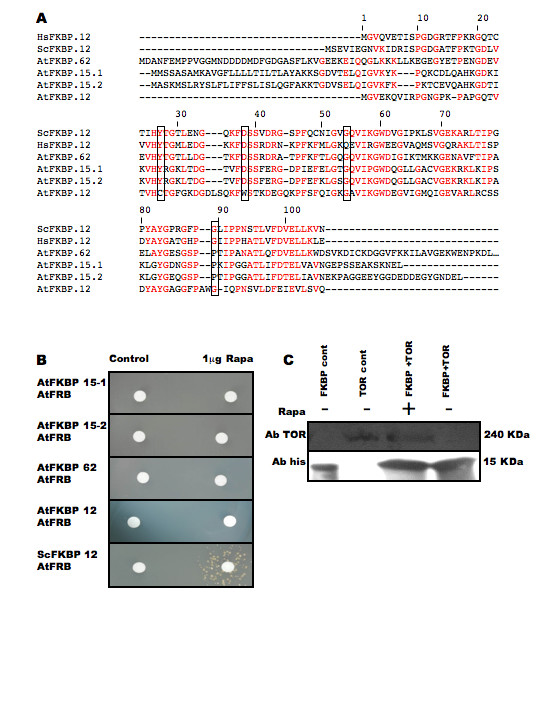
**AtFKBP are unable to complex with rapamycin and AtTOR**. A. Multiple alignment, using the Clustal program, of the AtFKBPs protein sequences with HsFKBP12 and ScFKBP12. Sequences are numbered according to HsFKBP12. Amino-acids involved in ternary complex formation are boxed. B. Two-hybrid analysis of the interaction between AtTOR FRB and AtFKBPs with ScFKBP as positive control. The yeast two hybrid strain AMY87-4 co expressing the GAL4(BD)::FKBP (were the FKBP used is indicated on the left of the picture) and the GAL4(AD)::AtFRB fusion proteins was spread on medium lacking adenine. Formation of the FKBP-rapamycin-FRB complex induces expression of the *GAL-ADE2 *reporter gene and is revealed by growth around the rapamycin disc (right). C. Pull down of native AtTOR with recombinant His-tagged ScFKBP. Track 1: Recombinant His-tagged ScFKBP. Track 2: Soluble Arabidopsis cell extract. Track 3: Recombinant His-tagged ScFKBP incubated with soluble Arabidopsis cell extract in the presence of rapamycin. Track 4: Recombinant His-tagged ScFKBP12 incubated with soluble Arabidopsis cell extract without rapamycin. Upper panel proteins were incubated with anti-AtTOR antibody (see methods). Lower panel proteins were incubated with anti-HisTag antibody.

The Arabidopsis genome contains 17 predicted FKBP-like proteins [27]. Comparison of Arabidopsis FKBP sequences shows that the closest relatives of ScFKBP12 are AtFKBP12, AtFKBP15-1, AtFKBP15-2 and the first FRB (FK506 and Rapamycin Binding) domain of AtFKBP62 (Fig. 1A). Some amino acids known to be involved in the formation of the rapamycin inhibitory complex [14] are absent from AtFKBP12 but are present in the other Arabidopsis FKBPs. This is the case of Tyr26 (numbered according to human HsFKBP12), Asp 38 and Gln 54 which are absent from AtFKBP12 but exists in AtFKBP15-1, AtFKBP15-2 and AtFKBP62. However, in these AtFKBPs a proline is replacing Gly89 which is known to be required for the complex formation (Figure 1A). This suggests that none of the plant FKBPs is able to engage into a TOR inhibiting complex with rapamycin. Menand et al [1] showed that, in a two-hybrid system, the FRB domain of AtTOR can bind to ScFKBP12. The same system was used to show that AtFKBP12, AtFKBP15-1, AtFKBP15-2 and AtFKBP62 are all unable to form a complex with the AtTOR FRB domain and rapamycin (Figure 1B).

### AtTOR can bind ScFKBP12 in the presence of rapamycin *in vivo *and *in vitro*

*In vitro *binding between ScFKBP12 and the native AtTOR protein was further investigated. To this end, recombinant ScFKBP12 was produced in *E.coli *as a fusion with a polyhistidine track and its binding to AtTOR was examined by pull-down experiments in the presence of rapamycin. Given the extremely large size of AtTOR, recombinant protein production would be difficult to perform. Hence the source of AtTOR was a proliferating Arabidopsis cell culture in which we previously observed a high level of expression of an AtTOR-GUS translational fusion [1]. An antibody directed against amino-acid 2341 to 2449 of AtTOR was raised in rabbits and used to detect the presence AtTOR in pull-down experiments.

Nickel-agarose bound ScFKBP12 was mixed with soluble Arabidopsis cell proteins with or without 10 μg/ml rapamycin and the resin washed before elution of ScFKBP12. Ni+ bound proteins were submitted to western blot analysis and His-ScFKBP12 and AtTOR were visualized using anti-His antibody and anti-AtTOR antibody, respectively. Figure 1C show that AtTOR can be detected at its predicted molecular mass (240 kDa) in a soluble protein extract from Arabidopsis cells (lane 2). When Ni+-agarose bound His-ScFKBP12 was mixed with Arabidopsis proteins in the presence of rapamycin, AtTOR can be detected together with His-ScFKBP12 in the resin eluate (lane 3), while in the absence of rapamycin only His-ScFKBP12 can be detected (lane 4). This shows that native AtTOR was retained to the column through a rapamycin-ScFKBP12 bridge and that binding of AtTOR to the ScFKBP12-resin did not occur in the absence of rapamycin.

### Expression of ScFKBP12 in Arabidopsis

**Figure 2:**
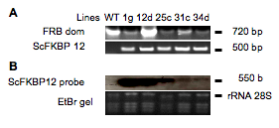
**Expression of ScFKBP12 in Arabidopsis transformed lines**. A. Upper panel: PCR amplification of the FRB domain of the AtTOR gene from plant DNA. Lower panel: PCR amplification of the ScFKBP12 transgene from plant DNA. B. Northern blot analysis of the 35S-ScFKBP12 transgene expression with ScFKBP12 probe (upper panel). RNAs were stained with EtBR (lower panel).

The above results show that ScFKBP12, rapamycin and AtTOR form a ternary complex *in vitro *and suggests that ScFKBP12 has the potential to inactivate AtTOR *in vivo *in the presence of rapamycin. This prompted us to test this idea by an experiment where ScFKBP12 would be expressed inside a plant cell. To this end, the coding region of ScFKBP12 was placed under the control of the constitutive CaMV 35S promoter and introduced into Arabidopsis (ecotype Columbia) through Agrobacterium-mediated transformation. About 20 independent primary transgenic plants were generated and lines homozygous for the transgene were selected using hygromycin resistance segregation. No obvious morphological phenotypes appeared in any of the selected lines. Insertion of the ScFKBP12 transgene was verified by PCR analysis. Northern blot analysis allowed to select five lines expressing the ScFKBP12 mRNA at different levels (Figure 2).

### ScFKBP12 expressing lines are susceptible to rapamycin

**Figure 3:**
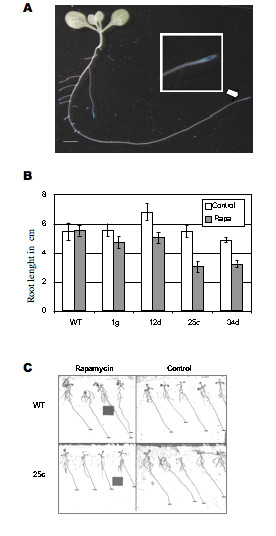
**ScFKBP transgene expression leads to rapamycin susceptibility**. A. GUS staining of an hemizygote for a T-DNA insertion within AtTOR [1] showing the expression of the AtTOR-GUS fusion protein, Scale bar 1 cm. Insert: close-up view of the primary root meristem. B and C, Primary root length measurment of 4 ScFKBP12 expressing lines compared with WT with 10 μg/ml of rapamycin (grey) or without rapamycin (white). A, 4 days after germination. B, 10 10 days after germination. The means of 20 roots are shown, with standard error of the mean indicated by the bars. B. Primary root length measurement. D. Picture of the 25 c line depicted in C. Scale bar 1 cm.

**Figure 4:**
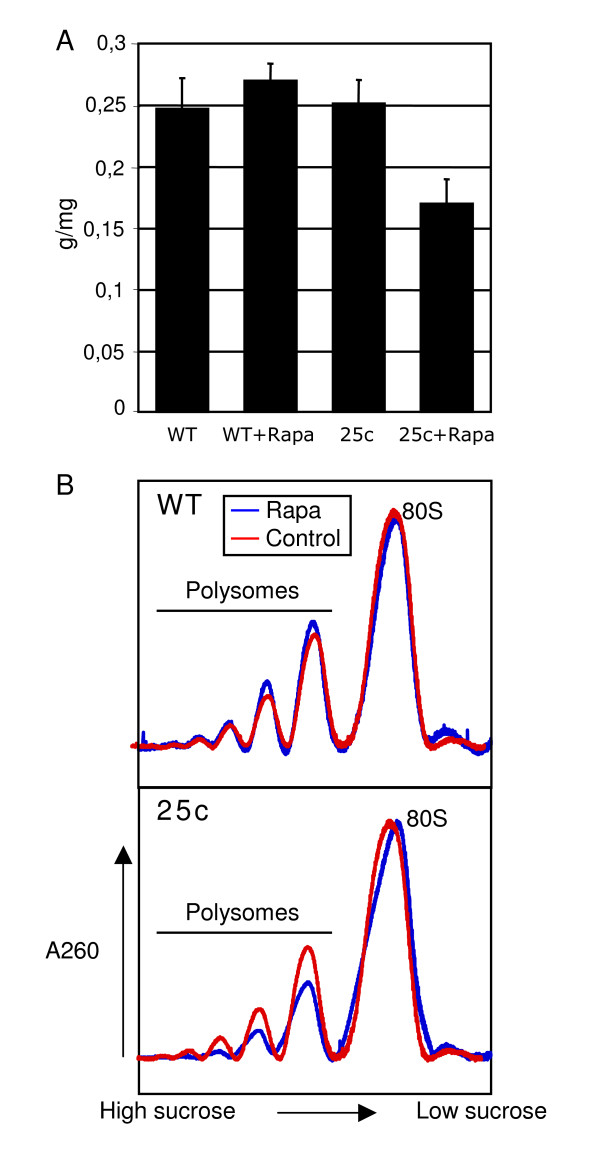
**Rapamycin inhibit growth of the ScFKBP expressing lines and reduce polysome accumulation. WT:control; 25c: transgenic line expressing ScFKBP12**. A. Effect of rapamycin on growth expressed as fresh weight per mg of seeds. Seeds were sown in liquid medium, incubated 48 h at 4°C, germination and grown under constant light during 10 days. Rapamycin was added at 10 μg/ml. B. Polysome profile from plantlets described in A. Polysomes were displayed on sucrose gradients and profiles recorded at 260 nm.

Expression analysis of the AtTOR gene fused to the GUS reporter gene showed that AtTOR is mainly expressed in meristems and particularly in the meristem of the primary root (Fig. 3A) [1]. The growth and architecture of the plant root system is very plastic and responds to changes in the availability of nutriments in the surrounding media. Therefore, for each transgenic line, sterile seeds were sown on vertical plates on synthetic media with or without rapamycin (10 μg/ml) using Col0 seeds as a control, and the growth of the primary roots was monitored. At ten days after germination, all transgenic lines displayed a significant growth retardation in the presence of rapamycin, while rapamycin had no effect on the primary root growth of the control plants (Fig. 3B). The line 25c show the highest reduction in primary root growth and a comparative increase in the length of secondary roots (Fig. 3C). This line was therefore selected for further analysis. This line does not display the highest expression of ScFKBP12 mRNA in leaves. The lack of a strict correlation between expression levels in leaves and rapamycin sensitivity is likely to be caused by variable transgene expression in the meristem, where AtTOR is present, depending on its genomic environment. In another experiment, 25 mg of Col0 control and 25c line seeds were allowed to germinate in liquid medium with or without rapamycin and fresh weight was recorded after 10 days. This shows again that overall growth of line 25c was reduced only in the presence of rapamycin. As one of the primary target of the TOR pathway is the protein synthesis machinery, plantlets from this experiment were further used to study the accumulation of polysomes. Although the polysome profiles of Col0 control plantlets grown with and without rapamycin were almost completely superposable, the profile of the 25c line displayed a lower accumulation of high molecular weight polysomes in the presence of rapamycin (Fig. 4). This strongly suggests that slower growth of the 25c line in the presence of rapamycin is a consequence of a reduced protein synthesis activity.

## Discussion

### ScFKBP12 binds AtTOR in the presence of rapamycin

All tested land plants appear to be resistant to rapamycin whereas *Chlamydomonas reinhardtii *is susceptible to rapamycin. This feature is likely to be due to mutations arising in the plant homologs of FKBP12 rather than in plant TOR proteins themselves. This work indeed shows that the native Arabidopsis TOR protein extracted from cultured cells can bind to the rapamycin-ScFKBP12 complex *in vitro*. These results support the *in vitro *interaction observed between a recombinant AtTOR FRB domain, rapamycin and human FKBP12 (HsFKBP) [25]. The rapamycin binding domain of TOR (FRB domain) is therefore functionally conserved among all eukaryotes, independently of the presence of FKBP proteins allowing ternary complex formation and inactivation of TOR. The function of this domain is still unknown but its wide phylogenetic structural conservation suggests that its role is independent of the binding of FKBP proteins. One likely hypothesis is that it binds a small molecule or protein that is structurally similar to rapamycin.

Given the diverse range of enzymatic activities that plants can display and the fact that rapamycin producing *Streptomyces *are soil borne bacteria, plant resistance to rapamycin might be the consequence of a detoxifying activity. The results presented here show that this is unlikely to happen since ternary complex formation in the presence of rapamycin can occur within a crude Arabidopsis protein extract. Moreover *in vivo *expression of ScFKBP12 can restore the activity of rapamycin in the transformed plants. This suggests that rapamycin is not efficiently detoxified in plant cells.

### *In vivo *sternary complex formation

Growth reduction in transformed plants expressing the ScFKBP12 protein occurred only in the presence of rapamycin. Since we have also shown that the AtTOR-rapamycin-ScFKBP12 complex can be formed *in vitro *but is dependent upon the addition of rapamycin, this strongly suggests that the observed decrease in growth is the consequence of an inactivation of AtTOR by rapamycin and ScFKBP12. However, we have previously shown that the knock-out inactivation of AtTOR by T-DNA insertion results in a complete halt of embryonic growth at an early stage [1] and it is known that rapamycin addition completely arrest growth in yeast and animal cells [4]. Therefore AtTOR inactivation by rapamycin in ScFKBP12 transgenic lines may be only partial. On one hand this could be due to inefficient translation, folding or stability of the ScFKBP12 protein or to limited diffusion of rapamycin in plant cells. On the other hand it is conceivable that AtTOR is mainly required during a short time window during embryogenesis and that further growth of the adult plant is only partially dependent of the TOR pathway, its inactivation leading thus to partial growth inhibition. The TOR pathway is known to control growth through ribosome biogenesis and translation [3,8] and rapamycin inactivation of TOR in yeast results in a drastically reduced accumulation of high molecular weight polysomes [28]. We show here that ScFKBP expressing plantlets displayed a reduced amount of high molecular weight polysomes, which correspond to actively translated mRNA, in the presence of rapamycin. Although the presence of the TOR protein seemed restricted to proliferative zones [1], inactivation of translation by rapamycin in the ScFKBP12 expressing lines was detected in the whole plant. It could thus be that AtTOR is present in all tissues but at a higher level in proliferative tissues where the demand for an active translation is higher. These results show that AtTOR is modulating translation in plants and that this control, and ultimately that of the growth process itself, is conserved through eukaryotes.

## Conclusion

This work shows that rapamycin susceptibility can be restored in plants by expression of an heterologous FKBP and that land plant rapamycin resistance is likely to occurs through evolution of the plant FKBP. The transgenic lines described in this work therefore represent the first available tools to inhibit TOR activity post-embryonically in Arabidopsis and will allow to further study the functions of the TOR signaling pathway in plants.

## Methods

### Arabidopsis Lines

The *Arabidopsis thaliana *cell suspension culture [29] was grown in sterile culture medium containing Murashige and Skoog salts (Sigma), 0.5 mM kinetin, 0.34 mM 2-4D, vitamins mix, (4 mM Nicotinic Acid, 1.26 μM Calcium Dpantothenate, 2.66 mM Glycine, 150 μM Thiamine-HCl, 110 μM Folic acid, 0.25 mM Pyridoxine-HCl, 20 μM Biotine and 28 mM Myo-inositol) and 3 % sucrose with pH 5.6. Cells in 100 mL of medium were incubated in a 250 mL conical flask and shaken at 125 rpm at 25°C in an orbital shaker under constant illumination (Infors, Massy, France). Every 9 d, subculturing was carried out by pipetting approximately 10 mL of the suspension (5% Packed Cell Volume) into 90 mL of fresh medium.

The Arabidopsis *tor*+/- mutants have been described previously [1]. Gus staining was performed as described with a 4 h incubation at 37°C [30]. Observations were performed with a Leica MZ FL3 binocular.

ScFKBP12 full ORF was amplified from pSBH1 [31] with primers 5'-CGGATATCATGTCTGAAGTAATTGAAGGTAAC-3' and 5'-GGACTGCAGCATGATGAGCTCTGCATCCGCCA-3'. The PCR product was digested with *Eco*RV and *Not*I and cloned under control of the CAMV35S promoter in pRT103 digested with *Xho*I, klenow treated and subsequently digested with *Not*I. The expression cassette was then moved into the unique *Asc*I site of the pGPTVHygro binary vector [32]. This construction was introduced by electroporation in *Agrobacterium tumefaciens *cells and transformation of Arabidopsis plants was carried out by the floral dip method [33]. The transformed plants were selected on solid medium with 30 μg of Hygromycin B and tested by PCR with the primer described above for the insertion of ScFKBP12. Control PCR of the AtTOR FRB domain was performed using primers: 5'-GCCATATGAGGGTTGCCATACTTTGGCATG-3' and 5'-GCAGATCCTTAGCTAGCTGTTTGTAATCCG-3'.

### Two-hybrid experiments

Two-hybrid experiments were performed according to [1] using *Saccharomyces cerevisae *strain SMY87-4 (MATa trp1-901 leu2-3, 112 ura3-52 his3-200 ade2 gal4 gal80Δ LYS2::GAL-HIS3 GAL2-ADE2 met2::GAL7-lacZ fpr1::hisG) which is resistant to rapamycin. This strain is a derivative of the two-hybrid host strain PJ69-4A in which the FKBP12 encoding gene is disrupted [34] and contain plasmid pTR17 (URA3) expressing a dominant rapamycin resistant allele of the TOR2 gene [35]. To generate GAL4(BD)::AtFKBP fusions Arabidopsis FKBPs were amplified from a cDNA library [36], using primers 5'-GGACTGCAGCATGATGAGCTCTGCATCCGCCATGAA-3' end 5'-GCAGCGGCCGCTCAAAGCTCATTCTTTGATTTCGC-3' for AtFKBP15-1, 5'-GGACTGCAGCATGGCCGACGAGATGAGTCTCCGTTA-3' and 5'-GCAGCGGCCGCTCATAGCTCATAGCTCGTCATTTCCATATCCC-3' for AtFKBP15-2, 5'-GGACTGCAGCATGGATGCTAATTTCGAGATGCCTCC-3' and 5'-GCAGCGGCCGCTCAACATATATCCTTCACACTGTCC-3' for AtFKBP62. PCR products were digested with *Pst*I and *Not*I and cloned in pBI880 digested using the same enzymes. For ScFKBP12, the full ORF has been excised from pSBH1 [31] using *Bam*HI. The fragment has been Klenow treated and cloned in pBI880 treated with the *Sma*I enzyme. For the AtFKBP12 construction (gift from JD Faure), the full ORF has been cloned between *Sal*I and *Not*I sites of pBI880.

After selection for the presence of the three plasmids, co-transformed yeast strains were grown overnight resuspended in top agar (0,7% in water) and spread on solid medium lacking leucine, tryptophan, uracil and adenine. 1 μg of rapamycin were deposed on Whatman paper discs, on the surface of the agar, and cells were incubated at 30°C for 5 days. Accession numbers; ATFKBP62: GenBank NM113429; AtFKBP15-2: Genbank NM124234; AtFKBP15-1: GenBank NM113428; ScFKBP12: GenBank M60877; AtFKBP12: GenBank NM125831; HsFKBP12: GenBank AAP36774.

### Pull down experiments

ScFKBP12 was amplified from pSBH1 using primers 5'-ATGGGATCCATGTCTGAAGTAATTGAAGGTACG-3' and 5'-GAGAAGCTTGTTGACCTTCAACAATTCGACG-3'. The purified PCR product was digested with BamHI and HindIII and cloned in the pET28a (+) vector (Novagen) digested by the same enzymes. *E.coli *strain rosetta (Stratagene) was used for protein expression. After IPTG induction, bacterially expressed proteins were loaded onto 1 ml of Ni-NTA agarose (Qiagen), and incubated for 30 minutes on ice. For pull down experiment, total soluble proteins from Arabidopsis suspension cells were prepared by grinding 1 g of cells in 10 ml of freshly prepared extraction buffer, (25 mM TrisHCl pH7.5, 10 mM NaCl, 10 mM MgCl2, 5 mM EDTA, 10 mM -mercaptoethanol, 1 mM PMSF, 0.2 mg/ml benzidine and 0.2 mg/ml leupeptin). The homogenate was centrifuged (15000*g*) for 15 min to remove insoluble material. Rapamycin was added to 1 ug/ml and 4 ml of this extract was loaded to the column containing recombinant ScFKBP12 followed by 2 h incubation on ice. The column was washed with 1 ml of Buffer A (50 mM Tris pH 7.4, Na2SO4 50 mM, glycerol 15%) and 1.5 ml of buffer A containing 30 mM imidazole. Proteins were eluted with 1,5 ml of 300 mM imidazole buffer and concentrated on Microcon YM50 (Millipore). For production of recombinant ScFKBP12, after loading of the bacterial extract, the column was washed in 4 ml buffer A and the proteins eluted as above.

### Western blotting and antibody production

Eluted samples were loaded on 4–12% SDS-PAGE gradient gels under reducing conditions. The resolved proteins were blotted onto Immobilon-P (Millipore, Bedford, MA), blocked in 5% skim milk, and probed with each primary antibody, followed by incubation with the alkaline phosphatase-conjugated secondary antibody. NBT/BCIP Western blotting detection reagents (Biorad) were used for detection. For production of the AtTOR antibody, a DNA fragment corresponding to amino-acids 2341 to 2449 of AtTOR was cloned into pET41 (Novagen) as a fusion with Glutathion-S-Transferase. Recombinant protein was produced in *E. coli *BL21 (DE3) and was used to generate antibodies in rabbits (Eurogentech). Anti-His antibody was from Amersham Biosciences.

### Northern blotting and polysome analysis

Total RNA was isolated with an RNeasy Plant Mini Kit (Qiagen, Tokyo, Japan) and displayed on Agarose gel containing formaldehyde (1.5%). RNA was transferred on Hybond-N+ (Amersham Biosciences) and cross-linked to the membrane. ScFKBP12 probe was amplified by PCR with pSBH1 using PCR DIG probe synthesis kit (Roche) Protocols and reagents for the chemiluminescent detection were according to the DIG luminescent detection Kit (Roche).

For polysome analysis, after stratification, 25 mg of seeds were grown for 10 days in 20 ml of liquid MS/2 medium containing 1% sugar at 25°C under constant illumination. Three hundred milligrams of seedlings were ground into a fine powder in liquid nitrogen and resuspended in 1 mL of lysis buffer, (100 mM Tris-HCl pH 8.4, 50 mM KCl, 25 mM MgCl2, 5 mM EGTA, 15.4 units/mL Heparin, 18 μM cycloheximide, 15.5 μM chloramphenicol, and 2% Triton X-100, 2 % Brij 35, 2 % Tween-40, 2 % NP-40, 2 % PTE, 10% Sodium Deoxycholate). Cell debris were removed by centrifugation at 7 000 rpm for 15 min at 4°C. Supernatants were loaded on 11 mL 0.8–1.5 M sucrose gradient made in 40 mM Tris-HCl pH 8.4, 20 mM KCl and 10 mM MgCl2. After centrifugation at 32 000 × g in a Beckman SW41 rotor for 150 min, gradients were fractionated with continuous monitoring of A260 in a Cary 50 spectrophotometer equipped with a 1 mm cell.

### Root growth measurement

Plant were sown *in vitro *on two times diluted Hoagland solution with 0.8% agar supplemented with 10 μg/ml of Rapamycin in DMSO. After 48 h at 4°C, plates were placed vertically under 16 h/8 h light/dark period at 23°C/18°C respectively. Root growth was monitored each day and measurements were processed with the NIH Image software.

## Authors' contributions

RS performed the analysis of transgenic plants, polysome analysis and prepared the figures, YL prepared recombinant protein and raised the antibody, BM performed genetic constructions, two-hybrid experiments and initiate plant transformation, CL and NJ helps with pull-down experiments, BM, CM and CR conceived the experiments, CM and CR wrote the manuscript.
